# Women’s experiences with using domperidone as a galactagogue to increase breast milk supply: an australian cross-sectional survey

**DOI:** 10.1186/s13006-023-00541-9

**Published:** 2023-02-07

**Authors:** Grace M. McBride, Robyn Stevenson, Gabbie Zizzo, Alice R. Rumbold, Lisa H. Amir, Amy Keir, Luke E. Grzeskowiak

**Affiliations:** 1 Adelaide Medical School, Robinson Research Institute, University of Adelaide , Adelaide, Australia; 2 SAHMRI Women and Kids, South Australian Health and Medical Research Institute, Adelaide, Australia; 3 Judith Lumley Centre, La Trobe University, Melbourne, Australia; 4 Breastfeeding Service, Royal Women’s Hospital, Parkville, Australia; 5 SA Pharmacy, SA Health, Adelaide, Australia; 6 College of Medicine and Public, Flinders Health and Medical Research Institute Flinders University, GPO Box 2100, SA 5001 Adelaide, Australia

**Keywords:** Domperidone, Breastfeeding, Cross-sectional studies, Galactagogues, Human milk, Lactation, Surveys

## Abstract

**Background:**

Domperidone is one of the most commonly utilised pharmacological galactagogues, with evidence of increasing use in clinical practice. However, the use of domperidone as a galactagogue remains controversial, with mixed evidence on safety and efficacy, leading to variable clinical practice recommendations. We sought to evaluate contemporary patterns of domperidone use and examine maternal experiences related to perceived safety and effectiveness.

**Methods:**

In 2019, we conducted an online, cross-sectional survey of Australian breastfeeding women to examine individual experiences related to domperidone use, in addition to perceptions of safety and effectiveness.

**Results:**

Among 1876 survey responses, 19% (*n* = 355) reported using domperidone. Domperidone use was significantly higher in women who were primiparous, gave birth preterm, delivered by caesarean section, had self-perceived low milk supply, and saw a lactation consultant. Nearly 20% of women commenced domperidone use in the first week postpartum (19%, *n* = 67). The median duration of use was six weeks (interquartile range 3–16 weeks). Maximum reported doses of domperidone used ranged from 20 mg/day to 160 mg/day. Half (*n* = 178, 50%) of women reported using a dose of 30 mg/day or less, 44% (*n* = 155) reported using a dose between 31 and 60 mg/day, and 6% (*n* = 22) reported using a dose greater than 61 mg/day. Nearly half of the respondents reported domperidone as ‘very’ or ‘extremely effective’ (45%, *n* = 161), with only 8% (*n* = 27) reporting it was ‘not at all effective’. Almost half (*n* = 172, 48%) of all women using domperidone reported side effects, including weight gain (25%), headaches (17%) and dry mouth (13%). Higher doses were associated with an increased likelihood of any side effects (≤ 30 mg/day, 38%; >31-≤60 mg/day, 48%, > 61 mg/day 73%; *P <* 0.004), with 31 (9%) stopping domperidone because of side effects.

**Conclusion:**

We identified widespread variation in domperidone utilisation patterns, with domperidone broadly perceived to be effective in increasing breast milk supply. Side effects associated with domperidone treatment were common, appeared to be dose-related, and were frequently associated with treatment cessation. These findings highlight the importance of improved clinical practice recommendations and generation of evidence from additional high-quality clinical trials evaluating the efficacy and safety of domperidone. More conclusive clinical trials are needed to determine the efficacy, as well as optimal dose and duration, of domperidone use.

**Supplementary Information:**

The online version contains supplementary material available at 10.1186/s13006-023-00541-9.

## Background

Low breast milk supply is one of the most common reasons women discontinue breastfeeding early [1–4]. Women seeking to increase their breast milk production often use galactagogues, defined as foods or medications thought to promote or increase breast milk supply [1, 5–8]. Domperidone, a dopamine receptor antagonist, is one of the most commonly reported galactagogues and is thought to improve breast milk supply by increasing serum prolactin [9, 10]. The treatment response window for domperidone use as a galactagogue is not well defined, however studies have shown it produces an initial prolactin increase within 60 min of treatment, with peak concentrations in the 4 to 5 days following, up to 800% of baseline levels [11]. Population estimates of use range from 5 in 100 women to 19 in 100 women in Australia and Canada, respectively, with several reporting significant increases in domperidone use during the postpartum period over the past two decades [12–14]. In Australia, domperidone is only available with a prescription.

Evidence supporting the efficacy and safety of domperidone during breastfeeding remains mixed. On the one hand, meta-analyses provide moderate quality evidence that the use of domperidone results in a modest increase in daily expressed breast milk volume of 90 mL/day in mothers who have experienced preterm birth [15]. In contrast, a recent Cochrane review of galactagogue use following term birth identified only low-certainty evidence that any pharmacological galactagogues, including domperidone, increase breast milk volume [16]. Differences in previous study methods are also reflected in large variability in existing clinical practice guidelines [17]. Regardless of the population being studied, previous trials have demonstrated significant heterogeneity with respect to outcomes [18]. This is partly attributable to differences in research methods, with trials investigating doses ranging from 30 mg/day or 60 mg/day, with the duration of treatment ranging from 4 to 28 days [18].

In addition to limited evidence on the efficacy of domperidone in lactation, there have been concerns about the safety of this medication. In 2014, the European Medicines Agency (EMA) issued warnings of QT prolongation and sudden cardiac death associated with domperidone use [19]. These warnings were mainly concerning risks in men over 60 years of age, when used as an antiemetic, but led to recommendations that a maximum of 30 mg/day be used for no more than one week in any adults [20]. However, the relevance of these warnings to breastfeeding women without clear risk factors for QT prolongation has been questioned [21].

Uncertainty regarding the efficacy and safety of domperidone has resulted in a lack of clear guidelines regarding its use during breastfeeding. This is reflected in the most recent guidelines from the Academy of Breastfeeding Medicine that, while stating domperidone may increase breast milk supply, do not provide any direct practice recommendations regarding optimal dosing regimens and treatment approaches [10]. This may lead to inconsistent prescribing practices and broad variations in dosing and duration of use [10, 22].

In light of agreed on practice recommendations and limited studies evaluating domperidone use during breastfeeding, we sought to understand real-world use and experiences based on a cross-sectional survey of Australian breastfeeding mothers.

## Methods

### Ethics

This study was approved by the Human Research Ethics Committee at the University of Adelaide (Approval number H-2019033934). The research was conducted according to the NHMRC National Statement on Ethical Conduct in Human Research 2007 (updated 2018) [23]. Survey responses were anonymous, where contact details were only required if participants chose to complete a follow-up interview as part of a separate study [24].

### Survey development and data collection

The survey consisted of two parts. Complete survey details, including a full copy of questions, have been published elsewhere [25, 26]. In brief, Part A included demographics (e.g. age, ethnicity, and education status), details of the most recent pregnancy and birth (e.g. parity, method of delivery, gestation at birth, plurality, attendance of antenatal classes, planned breastfeeding duration, receipt of breastfeeding support), galactagogues women had heard of, and their perceived safety of galactagogues to the mother and infant [26]. Part B questioned galactagogue use, including prescriber/source of recommendation, timing and duration of use, perceived effectiveness rated on a Likert scale from 1 (Not at all effective) to 5 (Extremely effective), and any side effects [25]. This paper utilises data from Parts A and B of the survey to evaluate women's experience of taking domperidone as a galactagogue.

The survey was piloted with a small cohort of consumers from the Australian Breastfeeding Association and Miracle Babies and academic experts in survey design, resulting in minor modifications before the final survey was launched. The survey was distributed through social media networks (i.e. Facebook, Twitter, email) of the Australian Breastfeeding Association, Miracle Babies, the Robinson Research Institute, and The University of Adelaide. The inclusion criteria for this survey were women living in Australia who were currently lactating or who had done so in the past. Participants were encouraged to share the survey and share links to the survey through their social networks. Therefore, the survey used non-probabilistic sampling. Participants were informed of the expected time to complete the survey (10 to 20 min), how data would be stored, and who would have access to information.

All investigators involved were named at the start of the survey, and contact details were provided. Contact details for several support services were given at the start of the survey if the questions caused participants any distress. Survey completion was voluntary, and no incentives were offered to participants. The survey was available online between 27 September 2019 and 12 December 2019, when sufficient responses were received. Adaptive questioning was used, where a series of more detailed questions concerning each galactagogue were only displayed if the participant indicated they had used that substance. A completeness check was not used before the survey could be submitted. Hence some 'incomplete responses' were submitted. Respondents were able to review and alter any responses before submitting. Unique site visitors, cookies or IP address tracking were not used.

Study data were collected and managed using REDCap (Research Electronic Data Capture) electronic data capture tools hosted at South Australian Health and Medical Research Institute [27, 28]. REDCap is a secure, web-based software platform designed to support data capture for research studies, providing 1) an intuitive interface for validated data capture; 2) audit trails for tracking data manipulation and export procedures; 3) automated export procedures for seamless data downloads to common statistical packages, and 4) procedures for data integration and interoperability with external sources. Only researchers named in the approved ethics application had access to the data.

### Statistical analysis

Data were cleaned and analysed using STATA 16 (StataCorp LP, College Station, TX). We searched for duplicate entries based on identical maternal characteristics provided in the entry section and removed the duplications. Individual incomplete questions were ignored during data analysis, rather than excluding the entire participant's data set. No weighting of the data was applied to adjust for representative samples.

The sociodemographic characteristics of women reporting domperidone use were first described and compared with those who did not report use, using Student's T-test for means and Pearson's Chi^2^ test for categorical variables. For those that did use domperidone, mean differences in perceived effectiveness were compared between groups using Student's t-test or one-way ANOVA. Median differences in duration of treatment were compared between groups using the Kruskal–Wallis test. Differences in those using specific doses or timing of use were compared using Pearson's Chi^2^ test, except for cell sizes less than five, where Fisher’s exact test was used instead. Statistical significance was defined as a *P* < 0.05. Graphical images were produced using GraphPad Prism version 9 (GraphPad Software, La Jolla, California, USA).

## Results

### Maternal demographics

Among 1876 women who responded to the survey, 355 (19%) reported using domperidone. Of the women who reported domperidone use, most (*n* = 314, 88%) used it in combination with other galactagogues, and only 41 women (12%) reported using domperidone alone. The characteristics of women who reported taking domperidone and those who did not are listed in Table 1. Overall, domperidone use was significantly higher in women who were primiparous, delivered prematurely, delivered by caesarean section, reported self-perceived low milk supply, and worked with a lactation consultant. Compared with those who didn't take domperidone, those who took domperidone reported higher levels of perceived safety for maternal health (mean ± standard deviation 3.9 ± 1.2 vs 3.5 ± 1.0; *P* < 0.001) as well as infant health (infant mean ± standard deviation 3.9 ± 1.2 vs 3.4 ± 1.0: *P* < 0.001).

**Table 1:** Sociodemographic and clinical characteristics of women using domperidone following their most recent birth

	**Took domperidone;** ***N* = 355**	**Did not take domperidone;** ***N* = 1521**	
	**n (%)**	**n (%)**	***P*—value**
***Characteristics of mothers and their youngest infant***			
** Mother's age at birth of youngest infant** **(years; mean ± SD)**	31.8 ± 4.6	31.4 ± 4.9	0.136
**State/Territory of child's birth**			0.040
** Australian Capital Territory**	10 (3)	78 (5)	
** New South Wales**	73 (21)	377 (25)	
** Northern Territory**	5 (1)	18 (1)	
** Queensland**	66 (19)	254 (17)	
** South Australia**	88 (25)	285 (19)	
** Tasmania**	12 (3)	30 (2)	
** Victoria**	70 (20)	337 (23)	
** Western Australia**	28 (8)	121 (8)	
**Primiparous**	205 (58)	672 (44)	< 0.001
**Multiple birth**	9 (3)	30 (2)	0.512
**Preterm birth**	74 (21)	141 (9)	< 0.001
**Caesarean section delivery**	162 (46)	455 (30)	< 0.001
**Infants age at survey**			0.237
** < 6 months**	110 (31)	447 (30)	
> **6 – ≤ 12 months**	79 (22)	290 (20)	
** ≥ 12 months**	163 (46)	765 (51)	
***Self-reported lactation difficulties and use of formula***			
** Perceived low breast milk supply**	327 (92)	920 (61)	< 0.001
** Saw a lactation consultant**	311 (88)	865 (57)	< 0.001
** Supplemented with infant formula**	251 (71)	305 (20)	< 0.001

### Prescribers

Participants reported that the majority of prescriptions for domperidone were provided by general practitioners (*n* = 271, 76%), followed by obstetricians (*n* = 72, 20%), midwives (*n* = 41, 12%), and neonatologists (*n* = 19, 5%). Approximately one in six (16%) women reported receiving a domperidone prescription from multiple prescribers.

### Doses

Maximum reported doses of domperidone used ranged from 20 mg/day to 160 mg/day. Half (*n* = 178, 50%) of women reported using a dose of 30 mg/day or less, 44% (*n* = 155) reported using a dose between 31 and 60 mg/day, and 6% (*n* = 22) reported using a dose greater than 61 mg/day. Maternal characteristics of each dose category are reported in Supplementary Table 1.

### Start period

Of all domperidone use, nearly 20% (*n* = 67) of women started using it in the first 7 days postpartum, and a further 38% (*n* = 134) started using it in the first four weeks postpartum. Women who started domperidone use earlier were more likely to report using higher maximum doses (see Supplementary Table 2).

### Duration of use

The duration of use varied from one week to greater than one year, with a median duration of 6 weeks (Interquartile range [IQR] 3 – 16 weeks; Supplementary Table 3). For women who were continuing domperidone use at the time of survey completion, 90% (*n* = 73/81) had been using domperidone for two or more weeks, where the median duration of use was 11 weeks (IQR 5 – 20). Duration of use was considerably longer for those reporting doses ≥ 61 mg/day (median 20 weeks, IQR 12 – 52 weeks; *P* < 0.001) compared to doses of ≤ 30 mg/day (median 4.5 weeks, IQR 2 – 11 weeks) and 31 – 60 mg/day (median 10 weeks, IQR 4 – 20 weeks).

### Side effects

Side effects are reported in Table 2. Nearly half of all women using domperidone reported side effects (*n* = 172, 48%), with 9% (*n* = 31) of all women using domperidone ceasing use due to side effects. Of those experiencing side effects, the most common side effect was weight gain (25%, *n* = 88), followed by headaches (17%, *n* = 59) and dry mouth (13%, *n* = 47). Thirteen (4%) women reported experiencing heart palpitations/racing heart with domperidone use. Of the women reporting side effects, 47% (*n* = 80) experienced two or more side effects. The frequency of stopping use because of side effects was highest in those reporting headaches (58%, *n* = 18), followed by weight gain (48%, *n* = 15) and dry mouth (28%, *n* = 9). There was no difference in cessation rates due to side effects between women with perceived low supply or no perceived supply problems (*P* < 0.699). Higher doses were associated with higher rates of side effects (≤ 30 mg/day, 38.2%; 31 – ≤ 60 mg/day, 48.4%, ≥ 60 mg/day 72.7%; *P* < 0.004, Chi^2^). The likelihood of medication cessation due to side effects increased as the dose increased (≤ 30 mg/day, 7.9%, *n* = 14; 31 – ≤ 60 mg/day, 9.0%, *n = *14; ≥ 60 mg/day 13.6%, *n* = 3; *P* < 0.000). A linear trend was observed between high perceived effectiveness and lower likelihood of women stopping domperidone due to side effects (*P* < 0.008).

**Table 2:** Side effects reported by women using domperidone according to the maximum dose used and whether treatment was ceased due to side effects

	**Maximum Reported Dose**				Ceased due to side effects*		
	** ≤ 30 mg/day; *N* = 178**	**31 – 60 mg/day; *N* = 155**	** ≥ 61 mg/day; *N* = 22**		**Yes; *N* = 31**	**No; *N* = 141**	
	**n (%)**	**n (%)**	**n (%)**	***P*-value**	**n (%)**	**n (%)**	***P*—value**
Any	74 (42)	82 (53)	16 (73)	0.004	31 (100)	141 (100)	< 0.001
Two or more	34 (19)	36 (23)	10 (45)	0.626	21 (68)	59 (42)	0.014
Weight gain	33 (19)	42 (27)	13 (59)	< 0.001	15 (48)	73 (52)	0.004
Headache	25 (14)	29 (19)	5 (23)	0.370	18 (58)	41 (29)	< 0.001
Dry mouth	18 (10)	24 (15)	5 (23)	0.133	9 (29)	38 (27)	0.012
Fatigue	12 (7)	18 (12)	1 (5)	0.272	7 (23)	24 (17)	0.011
Depression	9 (5)	7 (5)	4 (18)	0.054	6 (19)	14 (10)	0.004
Irritability	7 (4)	11 (7)	4 (18)	0.030	7 (23)	15 (11)	0.001
Nausea	8 (4)	4 (3)	1 (5)	0.509	2 (6)	11 (8)	0.316
Stomach cramps	7 (4)	6 (4)	1 (5)	1.000	5 (16)	9 (6)	0.004
Other	6 (3)	4 (3)	2 (9)	0.212	4 (13)	8 (6)	0.014
Heart palpitations /racing heart	4 (2)	7 (5)	2 (9)	0.140	6 (19)	7 (5)	< 0.001
Dizziness /fainting	3 (2)	7 (5)	2 (9)	0.075	3 (10)	9 (6)	0.077
Involuntary movements /jerking	3 (2)	1 (1)	0 (0)	0.711	1 (3)	3 (2)	0.307
Skin rash	1 (1)	1 (1)	0 (0)	1.000	2 (6)	0 (0)	0.007

^*^Total *n* = 172, where data are restricted to those who reported experiencing a side effect

### Perceived effectiveness

Forty-five percent of women felt domperidone was 'very' or 'extremely effective', 47% felt it was ‘slightly’ to ‘moderately’ effective, with only 8% reporting it was 'not at all effective'. Perceived effectiveness was not significantly different for women based on parity, method of birth, gestation at birth, or timing of commencement of domperidone (Table 3). Perceived effectiveness of domperidone was higher among those taking doses ≥ 61 mg/day, but the differences were not statistically significant (≤ 30 mg/day; mean ± standard deviation 3.3 ± 1.2, *n* = 178; 31 – 60 mg/day 3.2 ± 1.2, *n* = 155; ≥ 61 mg/day 4.2 ± 0.9 *n* = 22 *P* < 0.310, One-way ANOVA). Perceived effectiveness of domperidone was lower among those reporting a requirement to use infant formula due to supply difficulties, compared with those who did not require infant formula (3.2 ± 1.2 vs 3.6 ± 1.1; *P* < 0.001).

**Table 3:** Perceived effectiveness of domperidone use according to maternal and infant characteristics, rated on a Likert scale of 1 (not at all effective) to 5 (extremely effective)

	**n (%)**	**Mean ± SD**	***P*—value**
Infants age at survey			0.882*
< 6 months	110 (31)	3.2 ± 1.3	
> 6 – ≤ 12 months	79 (20)	3.4 ± 1.2	
≥ 12 months	163 (46)	3.3 ± 1.2	
Education level			0.711^#^
Completed secondary school	324 (92)	3.3 ± 1.2	
Did not complete school	31 (8)	3.3 ± 1.1	
Parity			0.943^#^
Primiparous	205 (58)	3.3 ± 1.2	
Multiparous	150 (42)	3.3 ± 1.2	
Plurality			0.794^#^
Multiple birth	9 (3)	3.2 ± 1.0	
Singleton	346 (97)	3.3 ± 1.2	
Gestation at birth			0.934^#^
Preterm	74 (21)	3.3 ± 1.2	
Term	281 (79)	3.3 ± 1.2	
Method of delivery			0.428^#^
C-section	162 (46)	3.3 ± 1.2	
Vaginal	193 (54)	3.4 ± 1.2	
Self-perceived breast milk supply			0.512^#^
Perceived low supply	327 (92)	3.3 ± 1.2	
No supply issue	28 (8)	3.5 ± 1.0	
Lactation support			0.517^#^
Saw a lactation consultant	311 (88)	3.3 ± 1.2	
Did not see a lactation consultant	44 (12)	3.4 ± 1.3	
Additional feeding requirements			0.001^#^
Required infant formula	251 (71)	3.2 ± 1.2	
Did not require infant formula	104 (29)	3.6 ± 1.1	
Dose used			0.310*
≤ 30 mg/day	178 (50)	3.3 ± 1.2	
31 – 60 mg/day	155 (44)	3.2 ± 1.2	
≥ 61 mg/day	22 (6)	4.2 ± 0.9	
Start period			0.412*
< 7 days	67 (19)	3.0 ± 1.3	
1 – 4 Weeks	134 (38)	3.3 ± 1.2	
> 4 Weeks	154 (43)	3.5 ± 1.1	

^*^ One-way ANOVA

^#^ Student's T-test

### Recommendations

Seventy-eight percent (*n* = 277) of women responded that they would use domperidone again. Eighty-two percent (*n* = 290) of women reported that they would recommend domperidone to a friend, whereas 18% (*n* = 62) would not recommend it. the most common reasons for not recommending domperidone were that it did not work (55%, *n* = 34), and because of side effects (40%, *n* = 25). Other reasons for not recommending domperidone use included that is was too expensive (3%, *n* = 2), and ‘other’ (13%, *n* = 8). The likelihood of recommendation domperidone to a friend increased significantly with higher perceived effectiveness (*P* < 0.000). Women who ceased domperidone use due to side effects were also less likely to recommend domperidone to a friend (*P* < 0.000).

## Discussion

In this large online cross-sectional survey, we identified wide variation in domperidone utilisation patterns, with domperidone broadly perceived to be effective in increasing breast milk supply. Side effects associated with domperidone treatment were common and appeared to be dose-related, with 1 in 11 women ceasing domperidone due to side effects. These findings highlight the importance of improved clinical practice recommendations and evidence regarding domperidone use in lactation.

The overall prevalence of domperidone use of 1 in 5 is higher than that reported in previous Australian studies showing 1 in 20 women using domperidone [12]. Our finding that approximately one third of women who gave birth preterm and a quarter who gave birth via caesarean section used domperidone and were more likely to be primiparous is similar to previous Australian and Canadian studies [12, 13]. Approximately 20% (*n* = 67) of women started using domperidone in the first week following birth, raising concerns that some women may be using domperidone before their breast milk supply has had adequate time to be established or before they have accessed professional lactation support. Additionally, 8% (*n* = 28) of women in this survey said they did not ever feel that they could not make enough milk for their infants, yet took domperidone. While we do not why they took domperidone in this instance, it may be related to underlying concerns about their ability to breastfeed successfully, which may or may not be shaped by prior experiences [24].

We identified significant variability in the maximum daily dose of domperidone, with doses ranging from 20 to 160 mg/day. Notably, the maximum dose used in published clinical trials is 60 mg/day [29, 30], with the maximum duration of treatment being four weeks [29]. Only a small number of respondents (*n* = 22; 6%) reported taking doses above 60 mg/day, which appears in stark contrast to international surveys and clinical audits where 67% received doses greater than 60 mg/day [13, 31]. Given the potential for greater side effects with higher doses, more evidence is needed on whether increasing doses above 60 mg/day is safe and effective. Considering the limited evidence regarding the optimal domperidone dose within mothers of preterm infants, and mothers of term infants, there is a clear need for more randomised controlled trials of domperidone to study efficacy and safety, as well as optimise dose and treatment durations.

Almost half of all women using domperidone reported experiencing one or more side effects. The most commonly reported side effect was weight gain, which has not been previously reported in clinical trials of domperidone during breastfeeding. Weight gain as a side effect of domperidone has been reported by one global survey on domperidone use, which found that 12% of women reported weight gain [31], compared to 25% of women in our survey. Other commonly reported side effects included headaches, dry mouth, and fatigue, which other studies have reported; however, it is noted that the assessment and evaluation of side effects in clinical trials remain limited [32]. We noted that side effects were more commonly reported as the maximum reported dose of domperidone also increased, which was also observed by Wan et al. [30], as was the increase in women ceasing domperidone at doses of 60 mg/day or greater.

One of the main controversies related to domperidone use is its potential to prolong the QT interval and increase the risk of ventricular arrhythmias or sudden cardiac death [19]. While we did not specifically ask about instances of diagnosed ventricular arrhythmias, several women reported feeling heart palpitations or a racing heart (*n* = 13, 3.7%), which can be an early warning sign of QT prolongation. This compares to a rate of 1.6% noted in a similar survey by Hale et al. [31]. Notably, the rate did not significantly differ between those who reported using domperidone, or another dopamine receptor antagonist, metoclopramide, which had no reports of QT prolongation in the survey by Hale [31]. Despite the potential seriousness of cardiac symptoms, only half of the women who experienced them reported stopping domperidone because of it.

Several large population-based studies of women who received domperidone postpartum (some including > 40,000 women exposed to domperidone [33]) have failed to demonstrate any statistically significant increased risk of ventricular arrhythmias or sudden cardiac death, suggesting the likelihood of such events is extremely rare. Despite this, it is important that women are informed about symptoms requiring further investigation. Even with these concerns about potential cardiac side effects, domperidone was still largely perceived as safe by women in this survey, which may demonstrate that these concerns for cardiac safety are not being communicated or that the perceived benefits outweigh the risks. This is supported by evidence from a qualitative study on a subset of women from this survey which found that galactagogue use is often underpinned by a strong determination to breastfeed, often resulting in women preferencing the potential benefits to their infant over potential risks to themselves [22, 24].

Nearly half of the women perceived domperidone as being very to extremely effective, with only 8% reporting it as being not at all effective, and the remainder finding it ‘slightly’ to ‘moderately’ effective. Of note, one study identified that one third of women experienced no improvement in breast milk volume at doses of 30 mg/day and 60 mg/day [30]. The potential for variability in treatment response is supported by a previous study demonstrating differences in prolactin response to domperidone based on parity [34], and differences in long-term breastfeeding outcomes based on maternal characteristics such as the method of birth and maternal BMI [35, 36]. However, we identified no differences in self-reported domperidone effectiveness based on maternal or infant characteristics, and could not assess impacts of maternal BMI in this study.

This study has several limitations. Participation in the survey was restricted to those living in Australia, and it is uncertain whether our findings are generalisable to other countries. Our overall population characteristics were broadly representative of Australian birth statistics known to influence breastfeeding outcomes such as maternal age at delivery, caesarean section, preterm birth, and prevalence of overweight [37]. The survey used non-probabilistic sampling which could lead to selection bias [38]. The survey only asked participants about the maximum dose of domperidone used rather than their starting dose, which may have differed. Further, we may have underestimated the actual duration of domperidone use, side effects and perceived effectiveness, as some mothers (23%) were continuing to take domperidone when completing the survey. We also did not ask women if they perceived any infant side effects as a result of domperidone use. Women self-reported whether they felt they could not make enough breast milk for their infants, we did not have data on actual milk production or underlying reasons for supply difficulties. Unfortunately, we did not ask women if they underwent an ECG to rule out any changes in QT prolongation following the self-reported side effects of heart palpitations/racing heart.

## Conclusion

We identified a significant increase in use of domperidone as a galactagogue in this breastfeeding population compared to previous Australian estimates, with large variation concerning how domperidone is being used and maternal perspectives of safety and effectiveness. Such variations in utilisation and outcomes are likely reflective of the limited evidence base regarding using domperidone as a galactagogue. These findings highlight the importance of improved clinical practice recommendations based on current evidence, as well as generation of evidence from high-quality clinical trials to determine the efficacy and safety of domperidone use.

## Supplementary Information

**Additional file 1: Supplementary Table 1.** Maximum dose of domperidone used during lactation according to maternal and infant characteristics.

**Additional file 2: Supplementary Table 2.** Postpartum timing of starting domperidone use by mothers in different groups.

**Additional file 3: Supplementary Table 3.** Median duration of use of domperidone in weeks according to maternal and infant characteristics.

## Data Availability

Data cannot be shared publicly because the ethics committee restricts secondary use of the data currently. Data are available on reasonably request made to The University of Adelaide Human Research Ethics Committee.

## Abbreviations

ANOVA: Analysis of variance
EMA: Europeans Medicine Agency
NHMRC: National Health and Medical Research Council
REDCap: Research Electronic Data Capture
SD: Standard deviation
