# Supplementary material for: Women’s experiences with using domperidone as a galactagogue to increase breast milk supply: an australian cross-sectional survey
Source: Int Breastfeed J. 2023 Feb 7;18:11. doi: 10.1186/s13006-023-00541-9 (PMC9903405; doi:10.1186/s13006-023-00541-9)
Supplement: Supplementary file 1 — Additional file 1: Supplementary Table 1. Maximum dose of domperidone used during lactation according to maternal and infant characteristics. [file 13006_2023_541_MOESM1_ESM.docx]

**Supplementary Table 1.** Maximum dose of domperidone used during lactation according to maternal and infant characteristics.

|  | **≤ 30** | **31 - 60** | **≥ 61** | ***P* - value** |
| --- | --- | --- | --- | --- |
| **N** | 178 | 155 | 22 |  |
| **Mothers age at birth** |  |  |  | 0.307* |
| **(years; mean ± SD)** | 31.3 **±** 4.5 | 32.2 ± 4.5 | 32.9 ± 5.9 |  |
| **Infants age at survey** |  |  |  | 0.127^#^ |
| **< 6 months** | 58 (33) | 49 (32) | 3 (14) |  |
| **> 6 – ≤ 12 months** | 37 (21) | 39 (25) | 3 (14) |  |
| **≥ 12 months** | 81 (46) | 66 (43) | 16 (73) |  |
| **Education level** |  |  |  |  |
| **Completed secondary school** | 164 (93) | 142 (92) | 18 (82) | 0.129^#^ |
| **Did not complete school** | 11 (6) | 13 (8) | 4 (18) |  |
| **Parity** |  |  |  |  |
| **Primiparous** | 101 (57) | 94 (61) | 10 (46) | 0.320^ |
| **Multiparous** | 77 (43) | 59 (39) | 12 (55) |  |
| **Plurality** |  |  |  |  |
| **Multiple birth** | 4 (2) | 5 (3) | 0 (0) | 0.854^#^ |
| **Singleton** | 174 (98) | 150 (97) | 22 (100) |  |
| **Gestation at birth** |  |  |  |  |
| **Preterm** | 31 (18) | 42 (27) | 1 (5) | 0.014^#^ |
| **Term** | 146 (83) | 113 (73) | 21 (96) |  |
| **Method of delivery** |  |  |  |  |
| **C-section** | 80 (45) | 72 (47) | 10 (46) | 0.960^ |
| **Vaginal** | 97 (55) | 82 (53) | 12 (55) |  |
| **Self-perceived breast milk supply** |  |  |  |  |
| **Perceived low supply** | 159 (90) | 147 (95) | 21 (96) | 0.154^#^ |
| **No supply issue** | 19 (11) | 8 (5) | 1 (5) |  |
| **Lactation support** |  |  |  |  |
| **Saw a lactation consultant** | 149 (84) | 142 (92) | 20 (91) | 0.079^#^ |
| **Did not see a lactation consultant** | 29 (16) | 13 (8) | 2 (9) |  |
| **Additional feeding requirements** |  |  |  |  |
| **Required infant formula** | 113 (64) | 124 (80) | 14 (64) | < 0.001^ |
| **Did not require formula** | 65 (37) | 31 (20) | 8 (36) |  |
| **Start period** |  |  |  | 0.094^ |
| **< 7 Days** | 27 (15) | 36 (23) | 4 (18) |  |
| **1 – 4 Weeks** | 63 (35) | 64 (41) | 7 (32) |  |
| **> 4 Weeks** | 88 (49) | 55 (35) | 11 (50) |  |

* One-way ANOVA; ^#^ Fisher's exact test; ^ Chi^2^ test
