# Supplementary material for: Women’s experiences with using domperidone as a galactagogue to increase breast milk supply: an australian cross-sectional survey
Source: Int Breastfeed J. 2023 Feb 7;18:11. doi: 10.1186/s13006-023-00541-9 (PMC9903405; doi:10.1186/s13006-023-00541-9)
Supplement: Supplementary file 2 — Additional file 2: Supplementary Table 2. Postpartum timing of starting domperidone use by mothers in different groups. [file 13006_2023_541_MOESM2_ESM.docx]

**Supplementary Table 2** Postpartum timing of starting domperidone use by mothers in different groups

|  | **< 7 days** | **1 - 4 weeks** | **> 4 weeks** | ***P* - value*** |
| --- | --- | --- | --- | --- |
| **N** | n (%) | n (%) | n (%) |  |
| **Infants age at survey** |  |  |  | 0.147* |
| **< 6 months** | 19 (29) | 50 (38) | 41 (27) |  |
| **> 6 – ≤ 12 months** | 11 (17) | 28 (21) | 40 (26) |  |
| **≥ 12 months** | 36 (55) | 54 (41) | 73 (47) |  |
| **Education level** |  |  |  |  |
| **Completed secondary school** | 63 (94) | 122 (92) | 139 (91) | 0.843^#^ |
| **Did not complete school** | 4 (6) | 11 (8) | 13 (9) |  |
| **Parity** |  |  |  |  |
| **Primiparous** | 37 (55) | 83 (62) | 85 (56) | 0.439* |
| **Multiparous** | 30 (45) | 50 (38) | 68 (44) |  |
| **Plurality** |  |  |  |  |
| **Multiple birth** | 1 (2) | 4 (3) | 4 (3) | 0.912^#^ |
| **Singleton** | 66 (99) | 130 (97) | 150 (97) |  |
| **Gestation at birth** |  |  |  |  |
| **Preterm** | 12 (18) | 30 (22) | 32 (21) | 0.763* |
| **Term** | 55 (82) | 104 (37) | 121 (43) |  |
| **Method of delivery** |  |  |  |  |
| **C-section** | 29 (43) | 67 (51) | 66 (43) | 0.365* |
| **Vaginal** | 38 (57) | 65 (49) | 88 (57) |  |
| **Self-perceived low breast milk supply** |  |  |  |  |
| **Perceived low supply** | 64 (96) | 122 (91) | 141 (92) | 0.538^#^ |
| **No supply issue** | 3 (5) | 12 (9) | 13 (8) |  |
| **Lactation support** |  |  |  |  |
| **Saw a Lactation consultant** | 63 (94) | 122 (91) | 126 (82) | 0.015^#^ |
| **Did not see an LC** | 4 (6) | 12 (9) | 28 (18) |  |
| **Additional feeding requirements** |  |  |  |  |
| **Required infant formula** | 54 (81) | 109 (81) | 88 (57) | < 0.001* |
| **Did not require formula** | 13 (19) | 25 (19) | 66 (43) |  |
| **Dose used** |  |  |  |  |
| **≤ 30 mg/day** | 27 (40) | 63 (47) | 88 (57) | 0.094^#^ |
| **31 – 60 mg/day** | 36 (54) | 64 (48) | 55 (36) |  |
| **≥ 61 mg/day** | 4 (6) | 7 (5) | 11 (7) |  |

* Chi^2^ test; ^#^ Fisher's exact test
