# Supplementary material for: Women’s experiences with using domperidone as a galactagogue to increase breast milk supply: an australian cross-sectional survey
Source: Int Breastfeed J. 2023 Feb 7;18:11. doi: 10.1186/s13006-023-00541-9 (PMC9903405; doi:10.1186/s13006-023-00541-9)
Supplement: Supplementary file 3 — Additional file 3: Supplementary Table 3. Median duration of use of domperidone in weeks according to maternal and infant characteristics. [file 13006_2023_541_MOESM3_ESM.docx]

**Supplementary Table 3.** Median duration of use of domperidone in weeks according to maternal and infant characteristics.

|  | **n (%)** | **Median (IQR)** | ***P* - value*** |
| --- | --- | --- | --- |
| **Infants age at survey** |  |  | 0.001 |
| **< 6 months** | 110 (31) | 5.5 (2 - 11) |  |
| **> 6 – ≤ 12 months** | 79 (20) | 8 (3 - 16) |  |
| **≥ 12 months** | 163 (46) | 8 (3 - 20) |  |
| **Education level** |  |  |  |
| **Completed secondary school** | 324 (92) | 7.25 (3 - 16) | 0.991 |
| **Did not complete school** | 31 (8) | 5 (2 - 9) |  |
| **Parity** |  |  |  |
| **Primiparous** | 205 (58) | 8 (3 - 16) | 0.171 |
| **Multiparous** | 150 (42) | 6 (3 - 13.5) |  |
| **Plurality** |  |  |  |
| **Multiple birth** | 9 (3) | 8 (4 - 16) | 0.026 |
| **Singleton** | 346 (97) | 6 (3 - 16) |  |
| **Gestation at birth** |  |  |  |
| **Preterm** | 74 (21) | 6 (3 - 16) | 0.548 |
| **Term** | 281 (79) | 6.5 (3 – 16) |  |
| **Method of delivery** |  |  |  |
| **C-section** | 162 (46) | 7.25 (3 - 16) | 0.464 |
| **Vaginal** | 193 (54) | 6 (3 - 16) |  |
| **Self-perceived breast milk supply** |  |  |  |
| **Perceived low supply** | 327 (92) | 6 (3 - 16) | 0.311 |
| **No supply issue** | 28 (8) | 6 (3.5 - 12) |  |
| **Lactation support** |  |  |  |
| **Saw a lactation consultant** | 311 (88) | 8 (3 - 16) | 0.991 |
| **Did not see a lactation consultant** | 44 (12) | 5 (2 - 8.5) |  |
| **Additional feeding requirements** |  |  |  |
| **Required infant formula** | 251 (71) | 8 (3 - 16) | 0.149 |
| **Did not require formula** | 104 (29) | 5.5 (2 - 13) |  |
| **Start period** |  |  | 0.306 |
| **< 7 Days** | 67 (19) | 11 (4 – 20) |  |
| **1 – 4 Weeks** | 134 (38) | 6.5 (3 – 15) |  |
| **> 4 Weeks** | 154 (43) | 6 (2 – 15) |  |
| **Dose** |  |  | < 0.001 |
| **≤ 30 mg/day** | 178 (50) | 4.5 (2 – 11) |  |
| **31 – 60 mg/day** | 155 (44) | 10 (4 – 20) |  |
| **≥ 61 mg/day** | 22 (6) | 20 (12 – 52) |  |

*Kruskal-Wallis test
